# A comparative study on machine learning based algorithms for prediction of motorcycle crash severity

**DOI:** 10.1371/journal.pone.0214966

**Published:** 2019-04-04

**Authors:** Lukuman Wahab, Haobin Jiang

**Affiliations:** 1 School of Automotive and Traffic Engineering, Jiangsu University, Zhenjiang, China; 2 School of Engineering, Tamale Technical University, Tamale, Ghana; University of British Columbia, CANADA

## Abstract

Motorcycle crash severity is under-researched in Ghana. Thus, the probable risk factors and association between these factors and motorcycle crash severity outcomes is not known. Traditional statistical models have intrinsic assumptions and pre-defined correlations that, if flouted, can generate inaccurate results. In this study, machine learning based algorithms were employed to predict and classify motorcycle crash severity. Machine learning based techniques are non-parametric models without the presumption of relationships between endogenous and exogenous variables. The main aim of this research is to evaluate and compare different approaches to modeling motorcycle crash severity as well as investigating the effect of risk factors on the injury outcomes of motorcycle crashes. Motorcycle crash dataset between 2011 and 2015 was extracted from the National Road Traffic Crash Database at the Building and Road Research Institute (BRRI) in Ghana. The dataset was classified into four injury severity categories: fatal, hospitalized, injured, and damage-only. Three machine learning based models were developed: J48 Decision Tree Classifier, Random Forest (RF) and Instance-Based learning with parameter k (IBk) were employed to model the severity of injury in a motorcycle crash. These machine learning algorithms were validated using 10-fold cross-validation technique. The three machine learning based algorithms were compared with one another and the statistical model: multinomial logit model (MNLM). Also, the relative importance analysis of the attribute was conducted to determine the impact of these attributes on injury severity outcomes. The results of the study reveal that the predictions of machine learning algorithms are superior to the MNLM in accuracy and effectiveness, and the RF-based algorithms show the overall best agreement with the experimental data out of the three machine learning algorithms, for its global optimization and extrapolation ability. Location type, time of the crash, settlement type, collision partner, collision type, road separation, road surface type, the day of the week, and road shoulder condition were found as the critical determinants of motorcycle crash injury severity.

## 1. Introduction

Globally, injuries resulting from road traffic crashes are a significant cause of death and disability with a disproportionate number occurring in African countries. The World Health Organization (WHO) stated that upwards of 1.2 million people die each year on the world’s roads, nearly half of which are those with the least protection: motorcyclists, cyclists, and pedestrians [1]. Fast economic growth in low- and middle-income nations has been associated with a surge in motorization and road traffic injuries [2]. In Ghana, studies have shown that one of the leading causes of death and injury is road traffic crashes, most of which occur in rural areas [3].

In the predominantly rural northern region of Ghana, motorcycle riding has long been a widespread and low-cost means of transportation. However, motorcycle use has recently become increasingly popular in cities as an alternative economic mode of transportation in congested road networks [4]. In contrast with other automobiles, motorcycle users are more vulnerable and thus more susceptible to serious injury [5]. Motorcycles permit riders to travel a longer distance in case the motorcycle has an engine with higher horsepower. Moreover, motorcycles are environmentally friendly because they produce less emission, occupy smaller space, and provide an alternative means of transportation for lower-income commuters. Thus, motorcycles are eco-friendly, offer a flexible, convenient, and inexpensive means of transportation when compared with four wheelers automobiles with an internal combustion engine. Regardless of these merits of motorcycles, there is a rising in safety concerns about the usage of motorcycles [6]. However, with the economic and technological developments, the motorcycle as a mode of transportation will be gradually replaced by e-bikes and public bicycle sharing system [7]. In Ghana, the cumulative number of registered motorcycles and three-wheelers as at the end of 2012 stood at approximately 23% of total registered motor-vehicles [1]. The total figure of newly acquired and registered motorized-two-wheelers in Ghana rose sharply from 11.5% (4,908) of entire registered automotive vehicles in 1995 to 29% (32,751) in 2016 [8]. Power-two-wheelers crashes and their related injuries and deaths are significant problems of transport safety in Ghana and have seen an upsurge in recent years. Fatalities associated with motorcycle crashes in Ghana shot to around 17.9% in 2015 from 2% in 1995 and are presently 21.0%, second in rank to the fatalities of the pedestrians [9]. Motorcycle crashes regularly occur on shared highways where motorcyclists take unusual and perilously riding behaviors. These riding behaviors include aggressive diverging, over speeding, riding in wrong-direction, unlawful lane changing, and wrong overtaking. These perilously riding behaviors of motorcyclists can lead to increasing the chance and level of severity of motorcycles involved in road traffic crashes [10].

Traffic safety is a global issue that is progressing at an alarming rate. It severely affects developing, as well as developed countries. There is an extensive and detailed body of work relating to two-wheeled motorcycle crashes and safety in developed nations; there are only a handful of studies involving rapidly emerging economies in Africa [11].

The current studies on road traffic crashes in Ghana [4,12–18] have focused primarily on the analysis of fatal crashes, and those involving pedestrians. However, where attempts have been made to study motorcycle crashes specifically [4,19–22], the focus has typically been on helmet usage and commercial motorcycle operations without consideration of the factors that influence crash severity. These studies have therefore created a knowledge gap that needs to be filled.

Iranitalab and Khattak [23] specified in their study that the modeling techniques that had been employed throughout the years to study modeling and prediction of road traffic crash severity can be grouped into two categories. These are statistical models, and data mining techniques.

Statistical models require a predefined mathematical form between dependent and independent variables. Additionally, they can be negatively influenced by missing values and outliers in the dataset. By contrast, machine learning models are non-parametric tools good at handling outliers and missing values. They are simultaneously able to hand-picked the supreme significant explanatory variables to classify the dependent variable [24]. Furthermore, these two model types have diverse properties: statistical models provide good theoretical interpretability with explicit mathematics construction; whereas machine learning techniques employ a “black box” tactic to forecast crash severity and often lack a reasonable explanation of the model. Compared to statistical models, machine learning methods are more accommodating with no or little presumptions for explanatory variables [25,26]. In addition, these machine learning based algorithms are more accomplished in dealing with outliers, noisy and missing data [27]. According to Tang *et al*. [27], the parametric models are excellent at the interpretation of theory with an explicit construction of calculation so that they can reveal direct and precise explanation to the relationship between motorcycle crash severity and interrelated variables than data mining approaches [28]. Nevertheless, statistical methods have the weakness that a number of these models presume that risk variables affect motorcycle crash severity is in a linear form which may not be the truth. This wrong presumption can lead to inappropriate inferences.

To address these drawbacks of statistical models, in this research machine learning based algorithms is proposed to predict motorcycle crash severity. In recent years, machine learning techniques have become contemporary methods in transportation safety research to identify the significant factors associated with crash severity [29–31]. They quickly explain the complex patterns associated with crash risk [32–35]. Unlike statistical models, machine learning based algorithms do not need any presumption mathematical functions that defined the relationship between endogenous and exogenous variables. They deal well with multicollinear explanatory variables and does treat reasonably nominal/discrete variables which are having more than two levels [36].

The primary objective of this work is to evaluate the application of a J48 decision tree classifier, random forest (RF), and instance-based learning with parameter k (IBk) model for predicting and classifying motorcycle crash severity. The performances of the models were assessed and compared to that of a multinomial logit model (MNLM). This study also identified and examined factors that are potentially significant to injury severity in motorcycle crashes. Identifying factors that significantly affect crash severity is one of the most critical tasks in traffic safety. Based on this, policy can be formulated to mitigate the number of fatalities and injuries resulting from crashes. The contributions of this research to motorcycle safety are threefold: firstly, to fill in the gap in the lack of application of machine learning in motorcycle crash severity analysis. It is an innovative study because the extensive review of existing literature revealed that this is the first time the J48 Decision Tree Classifier, RF and IBk models are employed to predict motorcycle crash severity. Secondly, investigating contributing factors associated with motorcycle crash severity in Ghana is under-researched; this study, therefore, contributes to the literature on motorcycle safety by fill in this gap. This research is the foremost of its type to study the outcome of injury from motorcycle crashes in Ghana; and lastly, this study utilized robust machine learning methods. Thus, the methods can easily produce nonlinear classification models with better generality.

## 2. Literature review

Over the years, numerous studies have applied a number of methodological techniques to explore the relationship between motor vehicle crash severity and its contributing factors [37]. Some of the methods that have been used include a binary logit models [38], binary probit model [39], ordered logit model [40], ordered probit model [41], bivariate probit model [42], multinomial logit [43], random parameter logit [44], artificial neural network [45], Bayesian [46,47], semi-nonparametric [48] (see [49,50] for comprehensive reviews of these models).

Furthermore, there is a substantial body of research available in which different classical statistical methods are used by researchers to investigate the factors that negatively impact motorcycle crash severity and improve the safety of motorcycle riders. The traditional starting modeling for motorcycle crash severity analysis is the binary logit/probit models [51–53]. Cafiso *et al*. [51] used logistic regression to develop a model for estimation motorcycles crash in Italy. The alternative to the binary logit model is the binary probit model which can deal with three restrictions of the logit model.

The statistics models evolved to consider multiple discrete outcome variables (for instance: fatality, hospitalized injury, injury, and damage only). In case of the multiple discrete outcome models, multinomial models are used if the injury outcome is more than two unordered categories. Geedipally *et al*. [54] investigated motorcycle crash severity in Texas, USA, using a multinomial logit model. Other numerous studies that used multinomial in their studies include Shaheed *et al*. [55] Jung *et al*. [56] and Savolainen and Mannering [57]. The multinomial logit model does not only account for the ordinal nature of the outcomes of the injury-severity level; it also does not impose limitations on the way variables affect outcome probabilities. However, the multinomial logit model is prone to the association of unobserved factors from the first injury-severity level to the second. Such association causes a violation of the model’s independence of irrelevant alternatives (IIA) property. If IIA is violated, the nested logit model ends this difficulty by putting together categories that have common unobserved effects into conditional nests [50]. Savolainen and Mannering [57] used a nested logit model to examine injury severities, to address the violation of IIA.

Recognizing the ranking nature of injury data (for example, ranging from fatal injury to hospitalized injury, to injury, to damage only) is essential in crash injury-severity analysis. To consider the ranking of the injury data, ordered response models have been extensively applied [50]. Chung *et al*. [58] utilized an ordered probit technique to examine injury severity in motorcycle used for delivery service and vehicle crashes in the metropolitan area of Seoul in Korea. Similarly, Blackman and Haworth [59] applied an ordered probit model to compare the crash risk and crash severity of larger scooters, motorcycles, and mopeds in Australia. Another study performed by Quddus *et al*. [60] used the same ordered probit model to assess the level of injury and powered-two-wheeler damage in Singapore to account for the nature of ordinal in the outcome of motorcycle crash injury. Rifaat *et al*. [61] assessed three ordered-discrete choice models to estimate the crash severity of powered-two-wheeler in Calgary, Canada. The results from their study revealed that partially constrained generalized ordered logit model, heterogeneous choice model, and ordered logit model yielded estimations that were very alike which shown the robustness of the models.

The classical crash severity models: discrete ordered (probit and logit models) and unordered models (such as the multinomial logit model) have a restriction that does not allow dependent variables to vary across individual outcomes. Meanwhile, each specific severity outcome responds totally in a unique manner to dependent variables and as such cannot be seen as fixed. Another major issue is that some of the variables that impact the level of severity of crashes are unobserved or are nearly unable to gather. If these unobserved variables (i.e., unobserved heterogeneity) are not addressed, might lead to the estimation of biased parameters and inappropriate inferences [62]. To counter the problem of unobserved heterogeneity, investigations on the level of severity of injury from motorcycle crashes in the current earlier works employed mixed logit (random parameters) [63–65], and bivariate [42]. They can deal with individual unobserved heterogeneity because of their ability to allow individual parameters to differ across observations [66].

Recently, several studies employed machine learning techniques to analyze and predict the crash severity of motorcycle crashes [26,29,31,67]. Anvari *et al*. [29] applied a classification and regression tree model to identify the most critical factors that influence at-fault motorcyclists involved in crashes. Kumar and Toshniwal [31] applied three classification algorithms to analyze factors that affect the severity of powered two-wheeler crashes and found the prediction accuracy of a classification and regression tree model was higher than that of a naïve Bayes classifier and support vector machine. Montella *et al*. [67] employed two of machine learning algorithms; rules discovery and classification trees analysis to analyze motorcycle crashes, to detect interdependence and differences amid crash characteristics The results provided an understanding for the development of motorcycle safety improvement strategies. They concluded that both classification trees and rules discovery were useful in producing meaningful insights about motorcycle crash characteristics and their interdependencies. Tavakoli Kashani *et al*. [26] utilized a classification and regression tree model to study factors affecting the crash severity of motorcycle pillion passengers. The study revealed that the predictive accuracy of their model showed considerable improvement compared to previous studies.

Theofilatos and Yannis [68] and Vlahogianni *et al*. [69] performed an extensive review of the existing literature on the safety of motorcyclist. Their study was concerning with collection of data, analysis methods, and contributory factors. They concluded that classical statistical methods dominate the field of motorcycle crash severity and a lack of application of machine learning methods is evident.

From the literature, several factors have been identified as contributing factors to motorcycle crash severity, including behavioral factors, as well as infrastructure-, vehicle-, and weather-related risk factors [69]. Several studies found the type, lighting condition, and time of the crash to be influential factors in predicting the injury severity [29,55,59,60,67,70,71]. Weather condition, collision partner, and traffic control were found to constitute another category of factors correlated with motorcycle crash severity [51,53,55,67,71]. Day-of-the-week was also a significant contributor to motorcycle crash injury severity [31,53,55,56,65]. Moreover, the settlement type: such as city, town, or rural; also affects motorcycle crash severity [26,29,31,61,67]. Previous studies discovered that the location of the crash: such as the intersection; was associated with the increasing severity of motorcycle crashes [55,61,70,72], while others found that the road description was related to injury severity [31,54,57,59,63,72].

In summary, prior studies mainly focused on econometric statistical (parametric) methods for the analysis of motorcycle crashes severity. So far, no research used J48 Decision Tree Classifier, RF and IBk models. Also, with the lack of study focusing on investigating contributing factors associated with motorcycle crashes in Ghana, motivates the topic of comparative study on machine learning based algorithms for prediction of motorcycle crash severity in the article.

## 3. Materials and methods

### 3.1. Dataset used

The data used in this study was obtained from the Traffic and Transportation Engineering Division of Building and Road Research Institute (BRRI) of the Council for Scientific and Industrial Research (CSIR), Ghana. The traffic crash data is electronically key-in into a Micro-Accident Analysis Package (MAAP) software developed by Transport Research Laboratory in the UK. The traffic crash data was initially gathered from the accident report file at the section of Ghana Police Service; Motor Traffic and Transport Unit (MTTU) [14].

The crash data recorded by BRRI has four types of motorcycle injury severities. The fatal Injury (F) is an injury where the casualty died within 30 days. The hospitalized Injury (H) is an injury where the victim of the crash was admitted at the hospital for more than 24 hours for medical attention. Whereas the not-hospitalized injury (I) is an injury where the victim of the crash was admitted at the hospital for less than 24 hours, and lastly damage only (D) is a crash where no death or no injury was recorded, but the vehicle involved was destroyed [3]. The data used in this study covers all motorcycle crashes occurring in Ghana from January 2011 to December 2015.

### 3.2. Tools

The Waikato Environment for Knowledge Analysis (WEKA) workbench was used to analyze the dataset with the data mining algorithms. It is an assembly of machine learning algorithms that are written in Java language and created by the University of Waikato in New Zealand. WEKA is an open-access software tool consisting of software that helps in functionalities of data mining such as preprocessing of data, visualization, feature selection, classification, regression, clustering, and association rules [73]. The functionality in WEKA is effortless because of its five built-in graphical user interfaces: Explorer, Experimenter, Knowledge Flow, Workbench, and Simple CLI. Explorer is used in producing the result metrics or statistics for the classification of a dataset. WEKA provides more than 100 classification’s algorithms, 75 pre-processing data algorithms, 25 algorithms for feature selection and evaluation and 20 algorithms for clustering and for finding association rules [74]. Version 3.8.3 of the WEKA toolkit was downloaded and installed on an HP equipped with 8 GB RAM, 2 GHz, a 64-bit operating system, and an AMD A6-7310 APU with integrated Radeon R4 Graphics to perform the experiments.

### 3.3. Preprocessing

Motorcycle crash data were obtained in the form of an Excel spreadsheet. Prior to the performing of the machine learning techniques, the data preprocessing was performed on the data set. Incomplete data such as data that which is lacking attribute values, missing values within the records were delete from the data set. Data that were inconsistent in names or codes with other recorded data in the data set were screen out from the records. Outlier analysis was performed. In WEKA a filter called Interquartile-Range was used to perform outlier analysis. This filter detects outliers and extreme values based on interquartile ranges. After this, a collection of charts presenting the visualizations of the value range of individual features was prepared to utilize the Weka graphical user interface. The charts permitted a coverage check to confirm that the likely range of values was present, and had the potential to detect outliers. The details of dataset used for this study is shown in S1 Dataset. The concluding list of the attributes and their descriptions are presented in Table 1. This motorcycle crash data comprises of all 8,516 crash records with 14 attributes.

**Table 1:** Attributes description.

Variables	Description	Injury severity level				Total
		Fatal (%)	Hospitalized (%)	Injured (%)	Damage (%)	
**Injury severity**		1952 (22.9)	3581 (42.1)	2500 (29.4)	483 (5.6)	8516
**Year**						
	1 = 2011	374 (23.8)	600 (38.1)	501 (31.8)	98 (6.2)	1573
	2 = 2012	401 (22.4)	701 (39.2)	570 (31.9)	116 (6.5)	1788
	3 = 2013	400 (25.5)	650 (41.4)	433 (27.6)	88 (5.6)	1571
	4 = 2014	398 (22.8)	800 (45.8)	468 (26.8)	79 (4.5)	1745
	5 = 2015	379 (20.6)	830 (45.1)	528 (28.7)	102 (5.5)	1839
**Location type**						
	1 = Not at a junction	957 (15.2)	2632 (41.8)	2337 (37.1)	371 (5.9)	6297
	2 = At junction	995 (44.8)	949 (42.8)	163 (7.3)	112 (5.0)	2219
**Settlement type**						
	1 = Urban	1010 (19.0)	2308 (43.5)	1699 (32.0)	293 (5.5)	5310
	2 = Village	799 (30.5)	1038 (39.6)	627 (23.9)	154 (5.9)	2618
	3 = Rural	143 (24.3	235 (40.0)	174 (29.6)	36 (6.1)	588
**Time of collision**						
	1 = Night	1135 (25.3)	2017 (44.9)	1149 (25.6)	187 (4.2)	4488
	2 = Day	817 (20.3)	1564 (38.8)	1351 (33.5)	296 (7.3)	4028
**Road description**						
	1 = Straight and flat	1780 (22.9)	3267 (42.1)	2283 (29.4)	438 (5.6)	7768
	2 = Curved and inclined	172 (23.0)	314 (42.0)	217 (29.0)	45 (6.0)	748
**Collision partner**						
	1 = car	842 (24.4)	1509 (43.7)	937 (27.1)	164 (4.8)	3452
	2 = HGV	156 (23.1)	283 (42.0)	196 (29.1)	39 (5.8)	674
	3 = Bus	267 (22.9)	490 (42.0)	340 (29.2)	69 (5.9)	1166
	4 = Motorcycle	549 (23.0)	1000 (42.0)	691 (29.0)	142 (6.0)	2382
	5 = Pickup	90 (17.9)	178 (35.3)	206 (40.9)	30 (6.0)	504
	6 = Bicycle	30 (17.5)	55 (32.2)	73 (42.7)	13 (7.6)	171
	7 = Other	18 (10.8)	66 (39.5)	57 (34.1)	26 (15.6)	167
**Road surface type**						
	1 = Tarred with potholes	655 (24.1)	1206 (44.4)	717 (26.4)	138 (5.1)	2716
	2 = Untarred	389 (21.0)	717 (38.7)	639 (34.5)	109 (5.9)	1854
	3 = Tarred and good	908 (23.0)	1658 (42.0)	1144 (29.0)	236 (6.0)	3946
**Day of week**						
	1 = Weekend	674 (24.2)	1238 (44.4)	740 (26.5)	137 (4.9)	2789
	2 = Weekday	1278 (22.3)	2343 (40.9)	1760 (30.7)	346 (6.0)	5727
**Weather condition**						
	1 = Clear	1737 (22.9)	3191 (42.1)	2228 (29.4)	432 (5.7)	7588
	2 = Other	215 (23.2)	390 (42.0)	272 (29.3)	51 (5.5)	928
**Road shoulder condition**						
	1 = Good	724 (24.7)	1282 (43.8)	778 (26.6)	145 (5.0)	2929
	2 = Poor	239 (23.0)	436 (42.0)	301 (29.0)	61 (5.9)	1037
	3 = No shoulder	989 (21.7)	1863 (40.9)	1421 (31.2)	277 (6.1)	4550
**Road separation**						
	1 = Median	528 (25.5)	924 (44.6)	529 (25.5)	93 (4.5)	2074
	2 = No median	1424 (22.1)	2657 (41.2)	1971 (30.6)	390 (6.1)	6442
**Traffic control**						
	1 = Signage	1006 (23.3)	1802 (41.8)	1270 (29.5)	232 (5.4)	4310
	2 = None	946 (22.5)	1779 (42.3)	1230 (29.2)	251 (6.0)	4206
**Collision type**						
	1 = Sideswipe	328 (27.3)	558 (46.5)	266 (22.2)	48 (4.0)	1200
	2 = Right-angle	200 (22.9)	366 (41.9)	263 (30.1)	45 (5.1)	874
	3 = Hit parked vehicle	116 (22.9)	212 (41.9)	148 (29.2)	30 (5.9)	506
	4 = Hit pedestrian	146 (23.0)	267 (42.0)	185 (29.1)	38 (6.0)	636
	5 = Rear end	669 (23.0)	1222 (42.0)	843 (29.0)	175 (6.0)	2909
	6 = Hit object off the road	99 (23.5)	178 (42.2)	124 (29.4)	21 (5.0)	422
	7 = Other	80 (22.9)	146 (41.8)	101 (28.9)	22 (6.3)	349
	8 = Animal	93 (23.2)	169 (42.1)	116 (28.9)	23 (5.7)	401
	9 = Head-on	131 (19.8)	290 (43.9)	202 (30.6)	38 (5.7)	661
	10 = Ran-off-road	90 (16.1)	173 (31.0)	252 (45.2)	43 (7.7)	558

### 3.4. Classifiers

In this research, the target (dependent) variable (motorcycle crash severity) is a one with four possible outcomes (fatal, hospitalized, injured and damage). Accordingly, the most suitable data mining functionality is classification. Classifiers are supervised machine learning algorithms that are used to classify dataset and deliver thought-provoking results. Classification techniques are predictive methods that are employed to forecast the classes of a target variable from measurements of one or more attributes (explanatory variables). The process of classification is categorized into three steps: firstly- Input has a defined set of known explanatory variables, secondly- Classifier to forecast the explanatory variables whose value is unknown, and lastly, output gives an unknown explanatory variables that have been determined by other known explanatory variables as a result of employing a classification algorithm [73].

In WEKA, several classifiers can handle classification problems. They are categorized into sub-packages such as Bayesian classifiers, decision trees classifiers, rules-based algorithms, functions algorithms, lazy classifiers, meta-learning algorithms and miscellaneous [74]. WEKA was employed for this study for four clear and exact reasons: firstly—it is a user-friendly tool because is having graphical user interfaces and secondly—it is an open software freely available under the general public license. Thirdly—it is very portable because of it is entirely executed Java programming language that can run on any modern computing platform and lastly, it comprises a complete set of data preprocessing and modeling approaches. In the succeeding sub-sections, a short explanation of the classification algorithms used in this work is presented.

#### 3.4.1. J48 decision tree classifier

J48 is a decision tree algorithm in WEKA. It is an open source of the C4.5 algorithm implemented in WEKA that creates a decision tree via information entropy. The method uses a “divide-and-conquer” approach to solve the learning problem from a group of independent instances [75]. J48 decision tree classifiers use explanatory variables to selects a target value of a new sample. The unique attributes are represented by the internal node of the decision tree; the branches between the nodes illustrate probable values that these variables can have in the observed samples, and the final node of the dependent variable is represented by the terminal nodes [76].

#### 3.4.2. Random forest

The RF approach employs the decision tree algorithm for parametrization, but it mixes a sampling procedure, a subspace technique, and an ensemble tactic to optimize the model building. Bootstrap is the name of the sampling method, which uses a random sampling tactic with replacement. The subspace technique also takes a random sampling method, but it assists in removing smaller subsets (i.e., subspaces) of variables [77]. The overfitting problem in decision trees is corrected in random decision forests by providing manifold trained decision tree algorithms for the testing stage. This property makes the RF a preferred over the regular decision trees algorithm [78].

#### 3.4.3. Instance-based learning with parameter k

IBk, also known as K-nearest neighbors (KNN) classifier, is one of the lazy-learning algorithms in WEKA. Lazy classifiers are learning strategies in which generalization of the training data is delayed until a query is made to the system. They differ from other classifiers that build a general model before receiving queries. The main benefit gained of utilizing a lazy classifier is that instead of estimating the target function on one occasion for the entire instance space, these methods can evaluate it locally and uniquely for every new instance to be classified. However, lazy classifier required both ample spaces to store the entire training dataset and times to produce the likelihoods [73]. The KNN classification algorithm is founded on the principle that instances inside a data will mostly exist nearby other similar instances. The label value of an unclassified instance can be classified by spotting the class of its closest neighbors in case the instances are marked with a classified label. The KNN locates the k closest instances to the query instance and finds its class by finding the single most recurrent class label [79,80].

#### 3.4.4. Multinomial logit model

The MNLM is a statistical method used to predict the probability of class relationship on a predicted variable constructed on several predictor variables. The expected variable in question is nominal and for which there are more than two categories while the predictor variables can be either dichotomous or continuous. The method is used to predict nominal response variables by representing the log odds of the responses are represented as a linear grouping of the explanatory variables. The MNLM is an upgrade version of binary logit regression that tolerates two or more categories of the outcome variable. Like binary logit regression, the MNLM applies maximum likelihood estimation to appraise the chance of categorical membership. MNLMs have restrictive assumptions of independence, normality, and multicollinearity [81].

### 3.5. Validation of the models

Witten et al. [82] asserted that the stratified 10-fold cross-validation technique is the acceptable method to validate the classifiers of a single and fixed sample data. The dataset is arbitrarily divided into ten sets. In each set, the class is characterized in roughly the same amounts as the entire dataset. Each section is held out in turn, and the learning algorithm is applied to the outstanding nine sets. Subsequently, the accuracy is computed on the holdout set. This process will mitigate any bias that can be generated by the holdout method which reserves a given quantity for testing and uses the remnant for training. Thus, the experiments in this study are performed using 10-fold cross-validation.

### 3.6. Performance metrics

The confusion matrix (contingency table) and its related performance measures: classification accuracy, precision, recall, true positive rate (TPR), false positive rate (FPR), and area under receiver operating characteristics curve (AUC)—were the parameters used to evaluate the accuracies of the classifiers used in this study. The bigger the figure of precision and recall the better the accuracy. A column in the contingency table indicates the predicted class instances, a row denotes the actual class instances, and the diagonal represents the accurate prediction. Therefore, the performance of a classifier can be visualized in the confusion matrix [82]. Table 2 shows the confusion matrix that is used to compute the above metrics.

**Table 2:** Confusion matrix.

		Predicted class	
		Yes	No
**Actual class**	**Yes**	True positive (TP)	False negative (FN)
	**No**	False positive (FP)	True negative (TN)

True positives (TP), as well as true negatives (TN), are correctly classified. A false positive (FP) is when the result is wrongly classified as “Yes.” A false negative (FN) is when the result is wrongly classified as “No” [82]. TPR measures the fraction of “Yes” that is correctly identified whereas FPR measures the fraction of “No” that are incorrectly classified. Precision is a measure that determines the exactness of a classification algorithm; a low precision indicates many FPs. Recall measures the completeness of a classifier; a low recall means several FNs. AUC is a vital tool for visualizing and evaluating classifiers. It can provide a more exact measure of classifier performance than scalar measures, such as classification accuracy [83]. An AUC value, near to 1, indicates outstanding performance while a value less than 0.5 indicates poor performance.

The formulas to calculate the metrics are shown in Eqs (1)–(4). (Eq 1), used for calculating TPR is also used to calculate recall.

$$TPR\;=\;\frac{TP}{TP+FN}$$ (1)

$$FPR\;=\;\frac{FP}{FP+TN}$$ (2)

$$Precision\;=\;\frac{TP}{TP+FP}$$ (3)

$$Accuracy\;=\;\frac{TP+TN}{TP+TN+FN+FP}$$ (4)

## 4. Experimental results and discussion

In this study, classification algorithms were applied to model the motorcycle crash severity. The injury severity attribute is used as the class attribute. This attribute takes four values as target values. The spreading of values in the dataset is presented in Table 1.

In total, 8516 motorcycle crash records were reported during 2011–2015. Of these, approximately 23% of the crash lead to fatal injury, 42% lead to hospitalized injury, 29% were classified as injured injury and, 6% as damage-only crashes. After preprocessing, the dataset is loaded as an Attribute-Relation File Format (ARFF) file into the WEKA data mining tool. Thirteen predictor variables (attributes) were utilized with the class variable to generate models to forecast the level of injury severity in a motorcycle crash.

Table 3 presents the performance metrics of all the four model types. Specifically, it shows the confusion matrix, TPR, FPR, precision, recall, AUC and classification accuracy obtained using 10-fold cross validation for each of the four classifiers. For each class, the confusion matrix reveals how instances from that class accepted the classifications used in this study. All correctly classified are in the diagonal of the contingency table. Hence, it is possible to inspect the matrix for errors visually.

**Table 3:** Comparison of classifiers performance metrics.

Classifier		Confusion matrix				TPR	FPR	Precision	Recall	AUC	Accuracy (%)
	**Actual**	**Predicted class**									
	**class**	**Fatal**	**Hosp**.	**Injured**	**Damage**						
	**Fatal**	852	893	179	28						
**J48**	**Hosp**.	77	3365	137	2	0.736	0.159	0.755	0.736	0.896	73.64
	**Injured**	30	530	1916	24						
	**Damage**	44	52	249	138						
	**Actual**	**Predicted class**									
	**class**	**Fatal**	**Hosp**.	**Injured**	**Damage**						
	**Fatal**	865	883	167	37						
**RF**	**Hosp**.	88	3346	147	0	0.739	0.157	0.757	0.739	0.902	73.91
	**Injured**	29	531	1913	27						
	**Damage**	34	49	230	170						
	**Actual**	**Predicted class**									
	**class**	**Fatal**	**Hosp**.	**Injured**	**Damage**						
	**Fatal**	872	878	171	31						
**IBk**	**Hosp**.	97	3341	143	0	0.737	0.158	0.755	0.737	0.902	73.71
	**Injured**	31	539	1906	24						
	**Damage**	39	53	233	158						
	**Actual**	**Predicted class**									
	**class**	**Fatal**	**Hosp**.	**Injured**	**Damage**						
	**Fatal**	583	1304	65	0						
**MNLM**	**Hosp**.	337	2565	679	0	0.520	0.287	0.544	0.520	0.704	52.04
	**Injured**	87	1132	1280	1						
	**Damage**	66	174	239	4						

Table 4 indicates the performance metrics by class for each class. The J48 classifier achieved an accuracy of 73.64%, with a precision of 0.849, 0.695, 0.772 and 0.755 for fatal, hospitalized, injured and damage, respectively. For the RF, the accuracy achieved was 73.91%, with a precision of 0.851, 0.696, 0.779 and 0.726 for fatal, hospitalized, injured and damage, respectively. For IBk, the accuracy was 73.71%, with a precision of 0.839, 0.694, 0.777 and 0.742 for fatal, hospitalized, injured and damage, respectively. Finally, using the MNLM, the accuracy was 52.04% with a precision of 0.543, 0.496, 0.566 and 0.800 for fatal, hospitalized, injured and damage, respectively.

**Table 4:** Comparison of performance metrics by class for each classifier.

Classifier	class	TPR	FPR	Precision	Recall	AUC
	**Fatal**	0.436	0.023	0.849	0.436	0.848
**J48**	**Hosp**.	0.940	0.299	0.695	0.940	0.898
	**Injured**	0.766	0.094	0.772	0.766	0.925
	**Damage**	0.286	0.007	0.719	0.286	0.913
	**Fatal**	0.443	0.023	0.851	0.443	0.851
**RF**	**Hosp**.	0.934	0.296	0.696	0.934	0.902
	**Injured**	0.765	0.090	0.779	0.765	0.934
	**Damage**	0.352	0.008	0.726	0.352	0.946
	**Fatal**	0.447	0.025	0.839	0.447	0.852
**IBk**	**Hosp**.	0.933	0.298	0.694	0.933	0.901
	**Injured**	0.762	0.091	0.777	0.762	0.934
	**Damage**	0.327	0.007	0.742	0.327	0.943
	**Fatal**	0.299	0.075	0.543	0.299	0.778
**MNLM**	**Hosp**.	0.716	0.529	0.496	0.716	0.640
	**Injured**	0.512	0.163	0.566	0.512	0.763
	**Damage**	0.008	0.000	0.800	0.008	0.584

Several characteristics of visualizations of the threshold curves are presented in Tables 3 and 4. The table indicates that for each classifier, the AUC was significantly higher than 0.5. However, that of the MNLM was the lowest. These AUC values indicate that the three classifiers have a superior ability to classify motorcycle crash severity correctly. Additionally, these three machine learning techniques out-performed the MNLM in predicting all injury severity classes. As the results indicate, RF was the most accurate classifier with the highest TPR, precision, and recall, and lowest FPR. Second to RF was IBk with slightly better performance metrics than J48.

One of the objectives of this empirical research was to evaluate the relative importance of explanatory variables in predicting the severity of motorcycle crashes. The gain ratio attribute evaluation method was used to determine the worth of each factor in predicting crash severity. The gain ratio is an extension of the information gain measure and used in the decision tree-based learning algorithm, C4.5. This measure overcomes the bias of information gain toward features with a large number of values by applying normalization [75]. Table 5 presents the relative importance ranking assigned by the gain ratio evaluator to each attribute implemented in WEKA concerning the class variable.

**Table 5:** Gain ratio of each attribute.

Attribute	Gain ratio
**Location type**	0.12236
**Time of crash**	0.01068
**Settlement type**	0.01012
**Collision partner**	0.00390
**Collison type**	0.00361
**Road separation**	0.00349
**Road surface type**	0.00224
**Day of week**	0.00222
**Year**	0.00179
**Road shoulder condition**	0.00171
**Traffic control**	0.00018
**Road description**	0.00004
**Weather condition**	0.00001

As shown in the table, the variables detected to have a stronger impact in determining the severity of motorcycle crashes are location type, time of crash, settlement type, collision partner, collision type, road separation, road surface type, day of week, year and road shoulder condition.

Lastly, the results herein are mostly consistent with those of previous studies from other regions. Concerning contributing factors, our study found that location type, time of crash, road description, day of week, traffic control, weather condition, settlement type, and collision partner had a similar impact in motorcycle crashes in other regions [53–56,59,67,71]. However, other contributing factors; such as road surface type and road shoulder condition; are not commonly examined in other studies, making these factors novel and significant in the Ghanaian situation.

## 5. Conclusions

Traffic crash analysis is one crucial task of road safety organization. Machine learning methods are non-parametric techniques that have been widely used in transportation research but are still relatively underutilized in motorcycle crash severity analysis. After a thorough literature review, we found a gap in the published studies on the methodology in motorcycle crash-injury severity research. Most research focuses on traditional statistical methods; this study focuses on machine-learning techniques.

Based on a five-year crash dataset, this study applied J48, RF, and IBk machine-learning techniques and the traditional MNLM to predict and classify motorcycle crash severity. Additionally, the study also determined the relative importance of factors that influence injury severity in a motorcycle crash. TPR, FPR, precision, recall, AUC, and classification accuracy were employed to appraise the performance of the models. The findings of this study revealed that the machine-learning methods outperformed the MNLM in classifying and predicting the crash severity. According to the cross-validated results, the best prediction performance was achieved by the RF model, followed by the IBk and J48 models. Although the MNLM showed satisfactory prediction performance, among the four techniques utilized in this experiment, its accuracy was the lowest.

Based on its performance, advantages (such as handling outliers and missing values) and ability to identify the most significant explanatory variable to predict the response variable, the results support machine learning techniques as an alternative model for predicting and classifying injury severity in motorcycle crashes. According to the most critical determinants of motorcycle crash injury severity identified in this research, a few countermeasure strategies are recommended to mitigate the severity of injuries in motorcycle crashes in Ghana. These safety strategies are the use of roadway facilities such as road signage and speed hump at junctions, enforcement of laws on red-light violations and speed limit. Others are enhancing visibility on roadway especially the use of street lighting and visibly road delineation and promote the use of reflective clothing to improve the conspicuity of motorcycle riders on the road.

## Supporting information

S1 Dataset The dataset (motorcyc_crash_severity) used for the study. (ARFF)
